# Surgical management of distal humerus gunshot fractures: descriptive case series

**DOI:** 10.1007/s00590-023-03611-0

**Published:** 2023-06-15

**Authors:** Ntambue Kauta, Alasdair Bott, Edgar Tafadzwa Majirija, Jean Pierre Du Plessis, Basil Vrettos, Sithombo Maqungo, Stephen Roche

**Affiliations:** 1 Cape Town, South Africa; 2 Southmead Hospital, Bristol, BS10 6NB UK; 3 Groote Schuur Hospital, University of Cape Town, Cape Town, South Africa; 4 Somerset Hospital, Cape Town, South Africa; 5 Vincent Pallotti Private Hospital, Cape Town, South Africa

**Keywords:** Gunshot Injury, Distal humerus fracture, Nerve injury, Vascular injury

## Abstract

**Purpose:**

The purpose of this study was to report our 5 years surgical experience and the rate of neurovascular injury following gunshot fractures of the distal humerus in a in level-1 Trauma Centre in South Africa.

**Methods:**

A retrospective case series of 25 consecutive adult gunshot injuries to the distal humerus. Demographic and injury data were extracted from clinical case notes and electronic operative records. Imaging archives were used to classify fractures according to the AO/OTA classification.

**Results:**

Twenty-five male patients, with mean age of 32-years-old, sustained gunshot injuries to the distal humerus. Eleven patients had multiple gunshots. Forty-four percent of patients underwent Computed Tomography Angiography (CTA), 20% had confirmed brachial artery injury. Limbs with vascular injury were salvaged with arterial repair and external fixation. Fractures were extra-articular in 20 cases (80%). Nineteen fractures were classified as highly comminuted. Nerve injuries occurred in 52% and were all managed expectantly. Only 32% of patients attended follow-up beyond 3 months.

**Conclusions:**

These are rare challenging injuries with high rates of neurovascular damage. This demographic of patients is poorly compliant with follow up highlighting the need for high-quality early care. Brachial artery injury should be excluded with CTA and can be managed with arterial repair and external fixation. All fractures in this series were surgically managed with conventional anatomical plate and screw fixation techniques. For nerve injury, we advocate expectant management.

**Level of evidence:**

IV.

## Introduction

Gunshot injuries to the distal humerus result in high energy transfer to a complex bony architecture which lies in a narrow soft tissue envelope, in close proximity to crucial neurovascular structures. They pose challenging clinical problems to treat in Orthopaedic surgery [1].

South Africa has high rates of civilian gunshot injuries. A substantial number of patients also have serious associated orthopaedic and visceral injuries, although as isolated injuries, they can still be life and limb threatening [2, 3]. Patient survival is dependent on emergency trauma care and early identification of vascular injuries [4].

In the longer term, the quality of fracture treatment and recovery from a peripheral nerve injury has a significant impact on functional outcome in a cohort who are often young males of working age, with high functional demands of their elbow joints [5, 6].

The purpose of this study was to report our 5-years surgical experience of gunshot injuries to the distal humerus and report on rates of neurovascular injuries and fixation techniques.

## Methods

We retrospectively screened our electronic operative database for all gunshot fractures of the distal humerus treated operatively in our department over a 5 years period (January 2014–December 2019). Inclusion criteria were: (i) Adult patients (aged 16 and older), (ii) Surgically stabilised gunshot fracture involving the distal one third humerus. Exclusion criteria: (i) Patients treated nonoperatively (ii) Fractures which also involved proximal ulna or radius (ii) Patients with insufficient documentation or imaging.

### Setting

Groote Schuur Hospital is a level-1 trauma centre in Cape Town, South Africa. It is a tertiary referral centre for complex orthopaedic elbow trauma and offers a full spectrum of reconstructive surgical capabilities including elbow arthroplasty and plastic surgery services. The hospital treats a high volume of gunshot injuries.

All patients were managed according to ATLS principles. Additional imaging modalities of the elbow were requested based on clinical examination and neurovascular status of injured limb. All patients were given antibiotic (Cefazolin) and tetanus prophylaxis as per our unit’s protocol. Low velocity injuries with small entry/exit wounds were treated as closed injuries. Routinely for posterior surgical approaches the ulnar nerve was decompressed and mobilised for fracture fixation but left in situ unless unstable. Nerve injury was managed expectantly, and nerve exploration was not routinely performed unless as part of the surgical approach used at time of skeletal fixation such as exposure. Peripheral Nerve Injury was classified as any weakness of motor function or sensory loss in an anatomical distribution.

Criteria for CT Angiography:

- Delayed capillary refill to hand and digits.
- Absent radial of ulnar artery on clinical examination.
- Suspicion of arterial injury.
- Pre-operative planning where soft tissue coverage was considered.

### Data collection

Clinical case notes and operative records were used to collect patient demographic data, details of peripheral nerve or vascular injuries, intra-operative findings, follow up compliance and complications.

Electronic imaging archives were reviewed to record the use of additional vascular imaging studies, operative fixation technique and progression to bone union. Plain radiographs and Computerized Tomography (CT) images were reviewed by two authors and bony injury patterns classified according to the Arbeitsgemeinschaft für Osteosynthesefragen/Orthopaedic Trauma Association (AO/OTA) classification and the presence of vascular injury assessed [7]. Where there was a disagreement, this was settled by consensus in a meeting with the senior author. Data analysis was performed using SPSS Version 27.

## Results

**Fig. 1:**
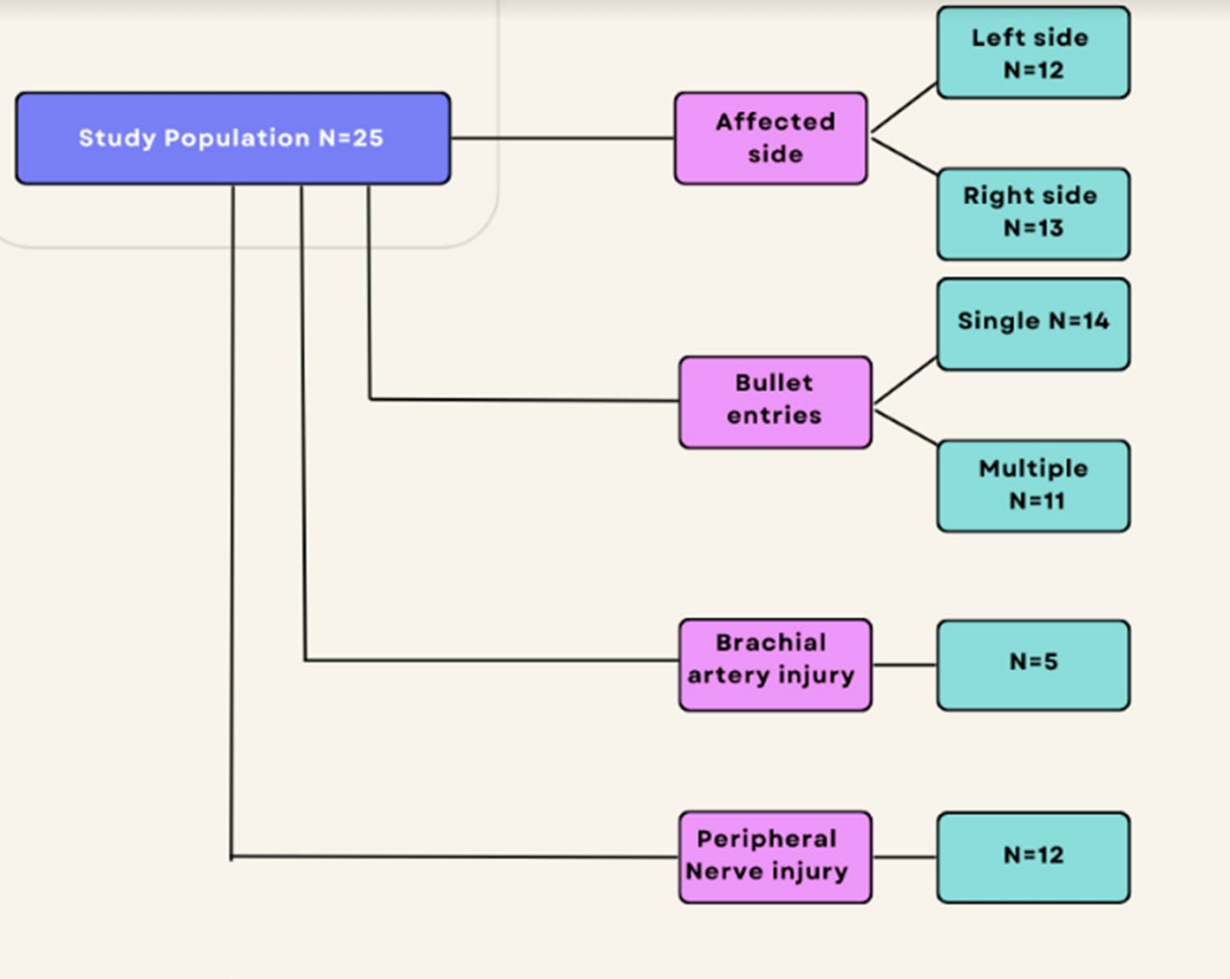
Affected side, single versus multiple gunshot injury, and rate of neurovascular injuries

Twenty-five patients met our inclusion criteria for the study. All were male, with a mean age of 32-years-old (range: 17–66 years, standard deviation 10.3 years). The left side was affected in 12 cases, and the right in 13. All injuries were suspected to have been caused by single, small calibre bullets. No shotgun injuries were seen. Isolated distal humerus injuries occurred in 14 patients, 11 patients had been poly-traumatised with multiple gunshots to neck, torso, abdomen or lower limbs (Fig. 1).

### Vascular injuries

Five (20%) patients sustained vascular injuries to the brachial artery. All these patients underwent emergency fracture stabilization with an external fixation at the time of vascular repair. Vascular repair was performed by direct repair or resection of affected artery section and vein graft grafting. Only one case required a fasciotomy at the time of vascular repair.

### Wound closure

Soft tissue injuries distant to surgical approach incisions were debrided and managed with primary closure in six cases, secondary healing in 18 cases and split skin grafting in one case. No cases were managed with free tissue transfer.

### Peripheral nerve injury

Twelve patients (48%) had documented peripheral nerve deficits with weakness of motor function or sensory loss in an anatomical distribution. There were seven radial nerve injuries of which six were complete, two complete ulnar nerve injuries, and two complete median nerve injuries. One patient had neurological fallout at initial assessment in both the ulnar and median nerve distributions.

**Fig. 2:**
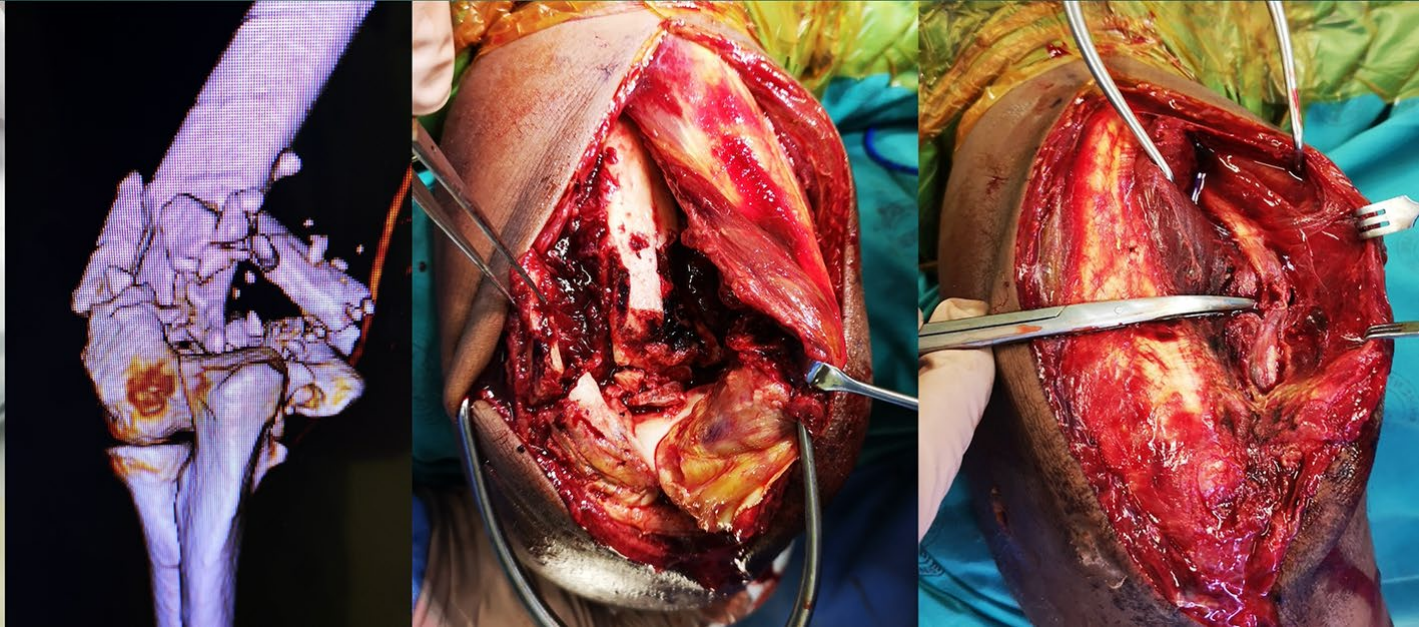
Ulnar nerve injury in patient with isolater A13.3 distal humeral fracture

In three cases of radial nerve palsy, the nerves were explored and found to be in continuity. In one patient with an ulnar palsy, exploration revealed the nerve to be contused over a distance of three centimetres but with an intact epineurium. A second patient with ulnar nerve palsy was explored as part of the surgical approach and found to have direct injury to the nerve at the level of medial epicondyle causing a central perforation through the nerve. (Fig. 2).

### Imaging

Plain radiographs were obtained for all patients. Eleven (44%) elbows underwent CT angiography for suspected vascular injury. A further three (12%) patients whose fractures had intra-articular extension underwent plain CT of the elbow to assist pre-operative planning. Four patients underwent a trauma series CT of head, C-spine, chest and pelvis without image capture of the affected elbow.

Based on the AO/OTA Classification, 20 of the fractures were extra-articular (type A), 17 of these were multi-fragmented type A3.3. One fracture was a partial articular type B, and four were type C fractures (Table 1).

**Table 1:** AO/OTA classification of distal humerus gunshot fractures

Location	AO/OTA class	Number (*n* = 25)	Percentage (%)
Extra-articular	13A1.2	1	4
	13A3.1	1	4
	13A3.2	1	4
	13A3.3	17	68
Partial articular	13B1.3	1	4
Complete articular	13C2	3	12
	13C3	1	4

### Surgical fixation

Mean time from injury to definitive fracture fixation was five days (range 0–19 days, standard deviation 4.5 days). Two patients had temporary external fixation following arterial repair followed by definitive fixation with single posterolateral locking plate. Four cases, which included three of the patients with brachial artery injury, had definitive treatment with external fixation. One patient was primarily treated in a backslab following initial debridement before undergoing definitive fixation at day 12.

**Fig. 3:**
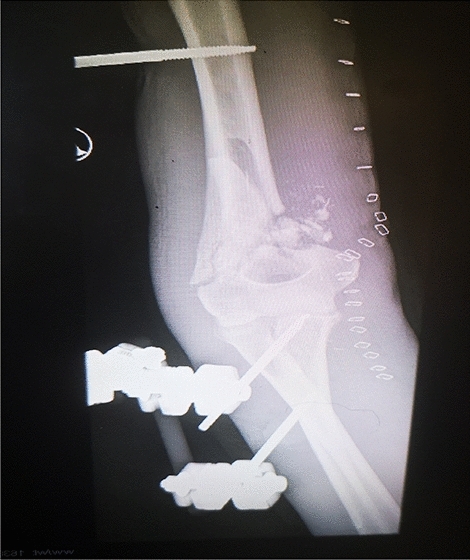
Gunshot distal humerus with brachial artery injury treated with temporary external fixator

**Fig. 4:**
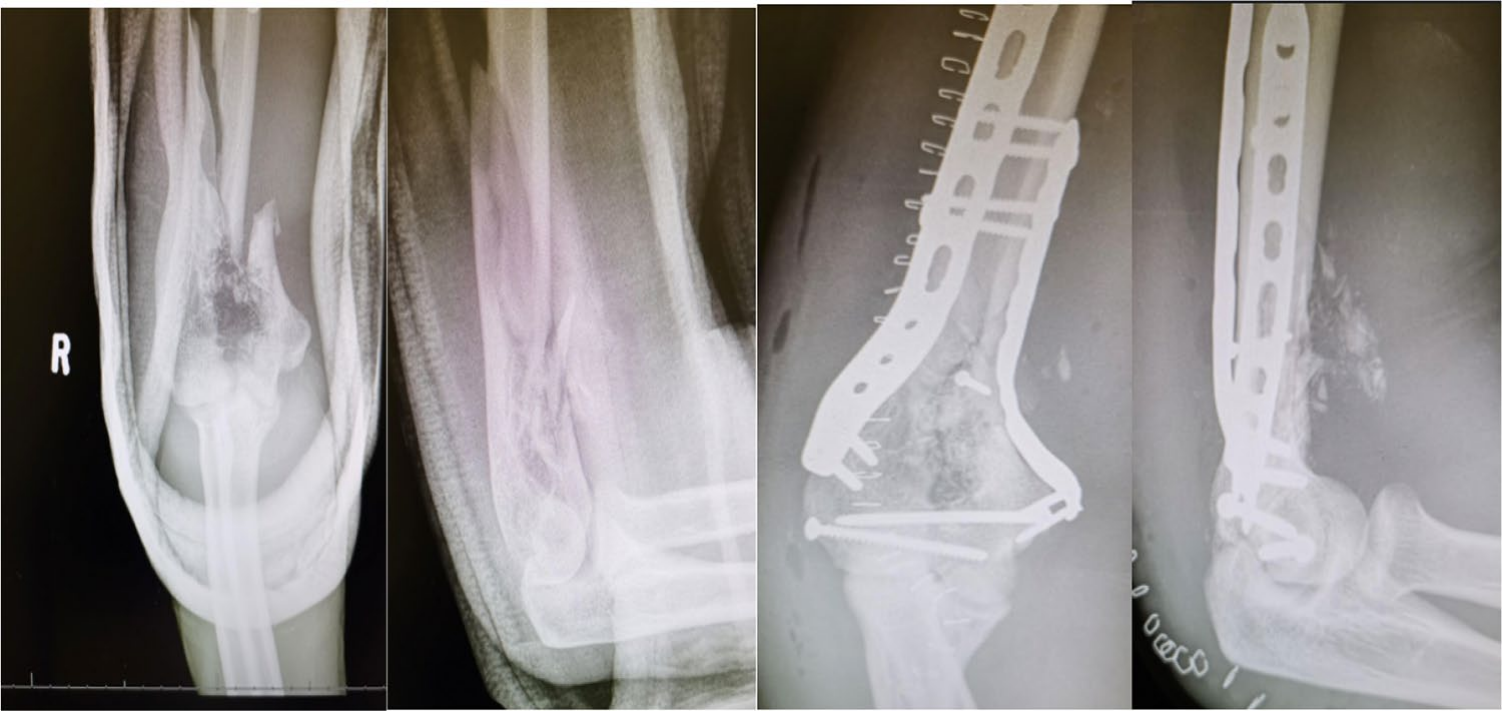
Pre-operative and post-operative radiographs of a gunshot distal humerus fracture treated with open reduction and internal fixation

Twenty-one patients were treated definitively as a single stage open reduction internal fixation with a bridging plate technique. Dual column fixation was the method used in seven cases. A single posterolateral plate was used in ten cases. Posterolateral plate with medial epicondyle screw fixation was used in two cases. Lateral column fixation in one case and screw fixation of the medial epicondyle in one case (Figs. 3, 4).

### Follow-up and complications

Eight patients did not return to any planned follow-up appointments, and 68% stopped attending follow-up beyond 12 weeks. Excluding external fixator removal, only one patient required early reoperation for wound coverage with split skin grafting 3 days after internal fixation. One patient was treated with oral antibiotics for superficial infection.

Of the eight patients who attended follow-up for longer than 3 months, six of the fractures had united. Three of these patients had persistent neurological fall out at their 12 weeks follow up. One patient required metalwork removal at 1 year and two represented with symptomatic non-union which required revision fixation within 2 years.

## Discussion

This study reports of 25 civilian gunshot fractures to distal humerus in a Level-1 Trauma Centre treated with contemporary techniques for over 5 years. This hospital has enormous experience of these kind of injuries due to gang related gun violence in the local catchment area. Similar to many other studies of civilian gunshot fractures, this cohort were young adult males of working age whose injuries appeared to have been generally caused by low-velocity firearms (8–10). 44% of this cohort were poly-traumatised having sustained multiple gunshots to other body regions.

Based on our protocol, 44% of patients with physical examination findings suggestive of possible vascular injury underwent CT angiography (CTA). It has been found that routine use of CTA may lead to overdiagnosis of arterial injury. Thus detailed clinical examination and selective use of CTA is a safe strategy supported in the literature [8, 9]. In many centres however, CT angiography is considered standard protocol when used in conjunction with image reformatting for planning of orthopaedic fixation.

Our study found 20% of patients with gunshot injury to the distal humerus also sustained injury to the brachial artery which required repair. These five patients had their fractures temporarily stabilized with an external fixator and immediate early vascular repair resulting in the salvage of all five limbs. This is in keeping with the literature on gunshot injuries to brachial artery which reports limb salvage rates of up to 97% with the techniques used in this study [10]. We recommend a low threshold for CTA in patients with gunshot injuries to the distal humerus.

Neurological deficits were recognised in 48% of this cohort, although of the five injured nerves explored as part of the surgical approach, only one nerve was seen to be directly injured, in which acute repair was not possible.

Expectant management of nerve injury is the treatment strategy employed in the majority of centres for good reason. Nerve injuries are most frequently the result of shock wave, heat and indirect trauma [11–14]. In one study of 41 upper limb nerve explorations following gunshot just 17% were found to have laceration to the nerve. However even in cases of direct trauma to a nerve buy gunshots, the zone of injury is difficult to define in the early post traumatic period and this makes acute nerve grafting an unsuitable strategy.

Most authors advocate the use of neurophysiology between 3 and 6 weeks post injury and it is accepted that approximately 70% of nerves in continuity will make functional recovery with expectant management within 3–9 months.

Recovery in some cases can be improved with decompression and neurolysis. Complete lacerations may be identified with neurophysiological testing. Patients with complete nerve lacerations, those without functional recovery and those with a positive Tinel’s test at site may only be suitable for excision and cable grafting once the zone of injury has stabilised.

Whilst there have been multiple reports of diaphyseal gunshot fractures, this study is the largest reported series of gunshot fractures affecting the distal humerus. Nineteen of the 25 were in the most severely multi-fragmented group based on the AO classification, we found the supracondylar region was the most commonly injured area and only five fractures had intra-articular extension without fracture of proximal forearm. Groote Schuur Hospital has experience in elbow arthroplasty, circular frame, bone transport and free bone transfer, yet none of these techniques were deployed. Only one elbow without associated vascular injury was managed definitively in a monolateral external fixator. All other fractures were fixed with conventional means of plates adhering to AO principles and bridging of areas of fragmentation with stable plate and screw constructs.

Gunshot wounds are a heterogeneous group of injuries. Our series comprises civilian gunshot injuries from low velocity weapons with small calibre bullets and they should not be regarded as all requiring radical tissue debridement. The features predictive of greater energy transfer are easily understood with an appreciation of terminal ballistics. Damage inflicted on tissues depends on the amount of kinetic energy possessed by the bullet, and the amount of energy transferred as it passes through tissues. Many of the techniques used in gunshot wounds to the arm are extrapolated from injuries to the tibia in that small wounds, treated as closed injuries, have been found to be an effective strategy, with low rates of infection and non-union [15].

### Limitations

There are several limitations associated with this study. The retrospective, single centre nature of the study and small sample size means that results may not be representative of all gunshot injuries to the distal humerus, particularly those resulting from high velocity military weapons.

The main limitation to this study is a high loss to follow-up and lack of clinical outcomes. This means that not all complications may have been captured. Although the majority of the patient cohort were local to our hospital, similar to other studies of gunshot injuries in South Africa, this patient cohort were poorly compliant with follow up. Previously we reported a 69% loss to follow-up after 16 weeks [16, 17]. Approximately 78% of demographic are unemployed and unable to afford transport to follow-up. Furthermore, there is approximately a five percent risk of death from another gunshot injury within 1 year [17]. Future studies would be improved with prospective data collection and incentivized follow up strategies to capture clinical outcomes and extent of neurological recovery.

## Conclusion

Gunshot fractures of the distal humerus rare challenging injuries with high rates of neurovascular damage. This demographic of patients is poorly compliant with follow up, and so good early care is imperative. Brachial artery injury should be excluded with CTA and can be managed with arterial repair and external fixation. Most other fractures can be managed with conventional fixation techniques. For nerve injury, we advocate expectant management.

## Data Availability

Not applicable.
